# Targeting of Apoptotic Cells Using Functionalized Fe_2_O_3_ Nanoparticles

**DOI:** 10.3390/nano5020874

**Published:** 2015-05-26

**Authors:** Moataz Mekawy, Atsushi Saito, Hiroaki Shimizu, Teiji Tominaga

**Affiliations:** 1 Department of Neurosurgery, Graduate School of Medicine, Tohoku University, 2-1 Seiryo-machi, Aoba-ku, Sendai, Miyagi 980-8575, Japan; E-Mail: tomi@nsg.med.tohoku.ac.jp; 2 Center of Materials Science, Zewail City of Science and Technology, Sheikh Zayed District, 12588, 6th of October City, Giza, Egypt; 3 Department of Neurosurgery, Aomori Prefecture Central Hospital, 2 1 1 Higashitsukurimichi, Aomori 0308553, Japan; E-Mail: satsushi2002@yahoo.co.jp; 4 Department of Neurosurgery, Graduate School of Medicine, Akita University, 1-1-1 Hondo, Akita 010-8543, Japan; E-Mail: hshim@nsg.med.akita-u.ac.jp

**Keywords:** functionalized nanoparticles (FNPs), fluorescently-labeled poly-caspase inhibitor (SR-FLIVO), apoptosis, brain diagnosis

## Abstract

Fe_2_O_3_ nanoparticles (NPs) have been synthesized and functionalized with SiO_2_ and -NH_2_ group, respectively. Conjugation to fluorescently-labeled poly-caspase inhibitor (SR-FLIVO) has been carried out for better cellular uptake studies of apoptosis arising from brain focal cerebral ischemia. Highest conjugation affinity to SR-FLIVO was found to be *ca.* 80% for Fe_2_O_3_-SiO-NH_2_ functionalized nanoparticles (FNPs). Tracking of SR-FLIVO conjugated functionalized nanoparticles (SR-FLIVO-FNPs) *in vivo* and *in vitro* has been carried out and detected using microscopic techniques after histochemical staining methods. Experimental results revealed that SR-FLIVO-FNPs probe could passively cross the blood brain barrier (BBB) and accumulated within the apoptotic cell. Optimization of SR-FLIVO-FNPs probe can effectively promise to open a new era for intracellular drug delivery and brain diagnosis.

## 1. Introduction

Apoptosis detection (denoted to a form of programmed cell death) has attracted much research in the past decade. It can be distinguished according to morphological, molecular, and biochemical changes occurred in the cell which is going to die. If apoptosis is diagnosed early and properly, there will be a high possibility for the prevention of its common contributing diseases. However, inappropriate regulation of apoptosis may play an important role for many pathological conditions in ischemia and stroke [1,2].

Previously, Annexin V has been addressed for apoptosis detection [3]; however, due to its very short life and the difficulty of reliably detecting it, the time point of assessment can be critical. In addition, Annexin V does not bind to all apoptotic tumor cells [4] and it also binds positively to normal and healthy bone marrow derived cells [5]. Furthermore, the inversion of phosphatidyl-serine may not be exclusively related to apoptosis and this adds to the background issues [6].

Distinguished signs for apoptosis are activation of caspases, DNA fragmentation, and membrane swelling [7]; therefore, caspase inhibitors play an important role in the early detection of apoptosis because the apoptotic cells have more active caspases than control cells.

Since the early reports of polycaspase inhibitor benzoxycarbonyl-Val-Ala-Asp-fluoromethyl ketone (Z-VAD-FMK) and its derivatives [8], they have attracted much attention. They are widely used because they can be bound specifically to the activated caspases, hence they can reflect detection of apoptosis. On the other hand, and as a progressive step, caspase inhibitors could be linked to organic dyes to facilitate the *in vivo* detection of apoptosis using enhanced fluorescence techniques [3].

Recently, nanotechnology based materials have been able to play an essential role not only in diagnosis but also in being an important platform for medical therapies. Several nanomaterials could be applicable for such purposes such as carbon nanotube [9], quantum dots [10], metallic [11], polymeric [12] and magnetic NPs [13] which have received considerable attention in the past decade to improve MRI diagnostics [14] taking into account their compatibility [15]. Thus, recent progress could lead to several models of magnetic NPs targeting for tumor imaging and therapy [16,17]. However, to date, the development of specific apoptotic-targeted NPs remains extremely challenging.

Several types of iron oxide based nanoparticles (NPs) have been used in the field of magnetic applicability. Only Fe_3_O_4_ and Fe_2_O_3_ had been approved by FDA to be used in clinical studies. However, Fe_2_O_3_ has better stability since Fe_3_O_4_ showed high tendency towards surface oxidation in the presence of oxygen and it is easily transformed to Fe_2_O_3_ even at room temperature [18,19,20,21].

The key factor that addresses the potential applicability of NPs in theranostics is their surface chemistry nature. Thus, surface functionalized nanoparticles are considered to be of high importance due to their small sizes with different surface natures and controlled penetration abilities which reflects their potential use in many biomedical applications such as *in vitro* cell separation, *in vivo* drug delivery, MRI contrasting agents and many others [22]. However, before discussing their applicability, the characteristics of nanoparticles should be examined carefully. Among those important characteristics, the ability to conjugate to different biomolecules, crossing the blood brain barrier (BBB), and accumulation inside the apoptotic cells for better modifications of the diagnosis techniques. In other words, it is important to examine the *in vivo* and *in vitro* cellular uptake of these nanoparticles to be applicable in the future as specific contrasting agents and/or drug delivery carriers.

In this study, we aim to synthesize a new probe for apoptotic cells detection based on functionalized Fe_2_O_3_ functionalized nanoparticles (FNPs). We have synthesized Fe_2_O_3_ core NPs shielded with silica-shell that is grafted with NH_2_ group for facile conjugation to the fluorescently-labeled poly-caspase inhibitor, sulforhodamine (SR-FLIVO) and for better molecular imaging studies of apoptosis arising from brain focal cerebral ischemia.

SR-FLIVO [3,23] is a red fluorescent probe that has the ability to diffuse across the cell through the BBB forming covalent bonds with activated caspases, and thus detects *in vivo* apoptosis. However, any un-reacted SR-FLIVO is leached outside the brain via the normal blood circulation. By considering the advantages of this molecular design which can allow the fluorescence detections based on hestochemical and TUNEL staining for the accumulation of SR-FLIVO-FNPs probe, one can predict that this probe can work effectively for *in vivo* brain imaging studies and open the era for inter and intracellular drug delivery. To the best of our knowledge, this is the first study to address such objectives using SR-FLIVO-FNPs probe.

## 2. Results and Discussion

### 2.1. Surface Morphology

**Figure 1:**
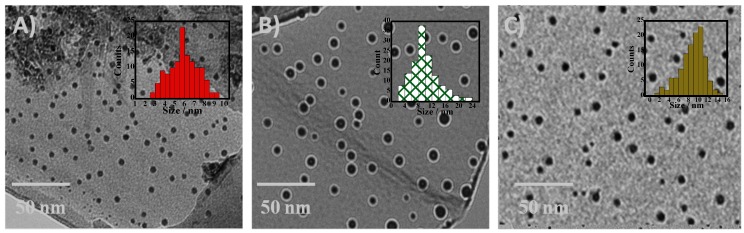
Transmission electron microscopy (TEM) and size distribution histograms for (**A**) Fe_2_O_3_; (**B**) Fe_2_O_3_-SiO_2_; and (**C**) Fe_2_O_3_-SiO-NH_2_ nanoparticles (NPs), respectively.

Transmission electron microscopy (TEM) experimental results (Figure 1) revealed that well resolved, mono-dispersed, and crystalline NPs and FNPs have been synthesized with average sizes of 6.3 ± 0.3, 10.8 ± 0.5, and 11.6 ± 0.6 nm for Fe_2_O_3_, Fe_2_O_3_-SiO_2_, and Fe_2_O_3_-SiO-NH_2_ NPs, respectively.

### 2.2. Conjugation to SR-FLIVO

**Figure 2:**
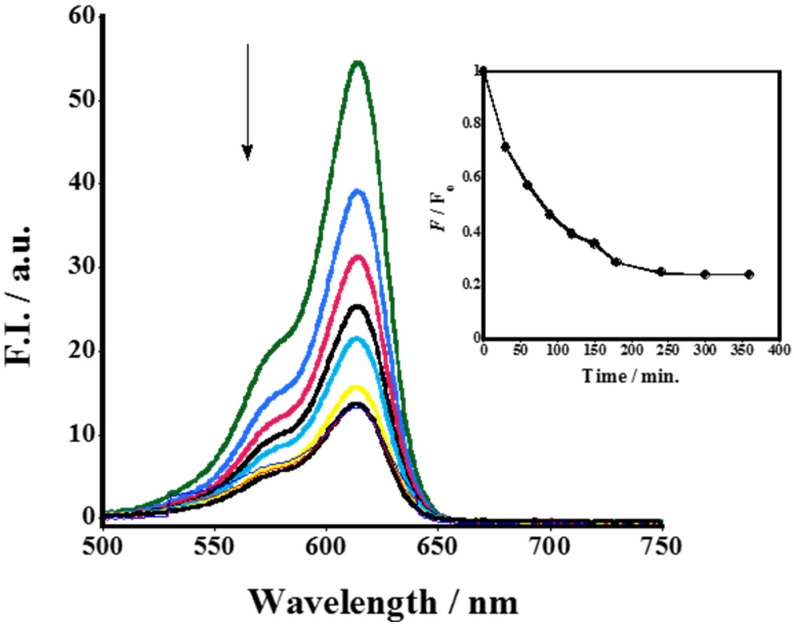
Time course fluorescence spectra measurement revealing the conjugation of Fe_2_O_3_-SiO-NH_2_ functionalized nanoparticles (FNPs) to fluorescently-labeled poly-caspase inhibitor (SR-FLIVO). The inset shows that the conjugation efficiency reaches *ca.* 80%.

**Figure 3:**
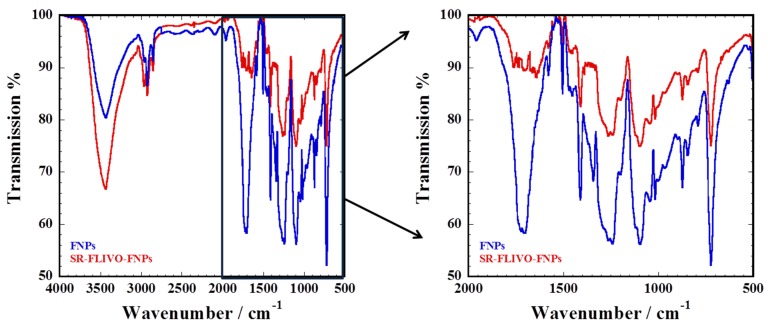
Fourier transform infrared spectroscopy (FTIR) spectra of FNPs (red) and SR-FLIVO-FNPs (blue).

The conjugation affinity which reflects the highest degree of FNPs surface coverage with SR-FLIVO was examined. Results shown in Figure 2 revealed that the conjugation affinity reached 80% for Fe_2_O_3_-SiO-NH_2_ FNPs. Time course fluorescence emission spectra was recorded and a remarkable quenching in the fluorescence intensity was noticed until reaching equilibrium. This could be ascribed to the formation of covalent bonding between the surface NH_2_ group of FNPs with the S=O group of SR-FLIVO [24,25,26]. This was confirmed by Fourier transform infrared spectroscopy (FTIR) spectra shown in Figure 3 which reveal the existence of a strong band at 1341 cm^−1^ which is ascribed to sulfonamide formation in SR-FLIVO-FNPs. Thus, it is considered among the candidate probes for apoptotic cells detection.

FTIR spectral results show several bands that could be assigned as: bands at 633 and 791 cm^−1^ which are attributed to the C–F stretching vibration in FLIVO, a strong band at 725 cm^−1^ which is attributed to the symmetric stretching vibration of Si–O–Si, a band at 845 cm^−1^ which is attributed to the wag N–H, a band at 875 cm^−1^ which is attributed to the stretching vibration of Si–O–H, a band at 1016 cm^−1^ which is attributed to the weak antisymmetric vibration of Si–O, a band at 1045 cm^−1^ which is attributed to the stretching vibration of Si–O–C arising from APTMS layer [27], and bands at 1095, 1237 and 1262 cm^−1^ which are attributed to the C–N stretching vibration of aliphatic amines. A strong band at 1341 cm^−1^ is attributed to sulfonamide stretching vibration that indicates the contribution of S=O group of SR-FLIVO in conjugation with free NH_2_ from FNPs. Bands at 1416 and 1458 cm^−1^ are attributed to the C–H bending vibration. A band at 1508 cm^−1^ is attributed to alkane H–C–H bending vibration. A band at 1580 cm^−1^ is attributed to the aliphatic N–H bending vibration. Weak shoulders appear at 1641, 1704, 1766 cm^−1^ that are believed to correspond to symmetric NH_3_^+^ deformation mode partly superimposed by CH_2_ bending and formation of labile H–bonding at FNPs [28]. A strong band with weak deformation appears at 1704 cm^−1^ and is attributed to the C=O stretching vibration at SR-FLIVO. A weak band appears at 1962 cm^−1^ and is attributed to the overtone bending vibration of aromatic C–H at SR-FLIVO. Bands at 2850, 2930 cm^−1^ are attributed to the symmetric and asymmetric stretching of CH_3_, respectively. A band at 3436 cm^−1^ is attributed to the stretching vibration of NH group.

### 2.3. TUNEL Staining

**Figure 4:**
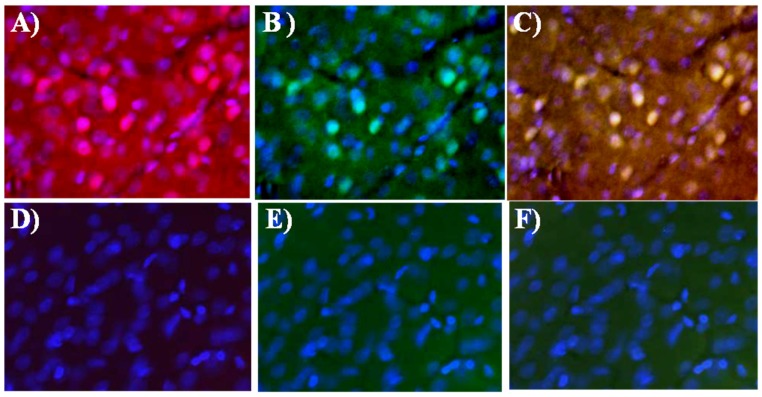
Fluorescence microscopy images after histochemical staining for ischemic lesion (upper raw) and non-ischemic lesion (lower raw) of SR-FLIVO-FNPs (**A**,**D**), terminal deoxynucleotidyl transferase dUTP nick end labeling (TUNEL) (**B**,**E**), and merged SR-FLIVO-FNPs/TUNEL staining (**C**,**F**) which show specific detection of apoptotic cells at the ischemic lesion in the rat brain after focal cerebral ischemia.

Figure 4 shows the enhancement of the fluorescence intensity of activated caspases at ischemic lesion, which is reflected with SR-FLIVO-NPs and terminal deoxynucleotidyl transferase dUTP nick end labeling (TUNEL) staining. In contrast, no enhancement was detected for the fluorescence intensity in the control (non-ischemic) side due to absence of activated caspases.

### 2.4. Intracellular Tracking

**Figure 5:**
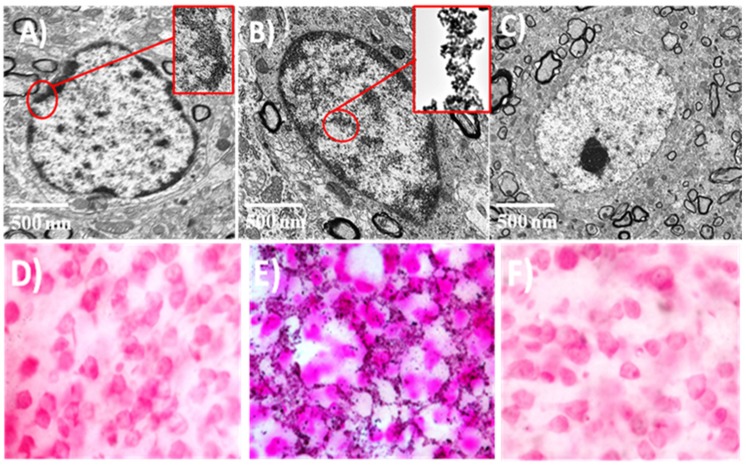
TEM micrographs for the apoptotic cells before (**A**) and after (**B**) injection of SR-FLIVO-FNPs. (**C**) control cell in non-ischemic lesion. Light microscope images after Prussian blue staining for (**D**) control rat brain after injection of SR-FLIVO-FNPs, (**E**) ischemic lesion of rat brain with localized SR-FLIVO-FNPs, and (**F**) non-ischemic lesion of rat brain after injection of SR-FLIVO-FNPs.

Cellular tissues were recorded using TEM after brain reperfusion of normal rats (*n* = 4), ischemic rats with and without injection of SR-FLIVO-FNPs (*n* = 4 for each group). Figure 5A shows the apoptotic cell before injection of SR-FLIVO-FNPs revealing surface blebbing which is considered a specific pattern of apoptosis. This is due to a deep cytoskeleton rearrangement, causing progressive changes in cell shape such as swelling and organelle distribution [29,30]. TEM micrographs showed that the SR-FLIVO-FNPs could passively crossing the BBB and deposited inside the apoptotic cells as shown in Figure 5B. This finding could also be confirmed using Prussian blue staining as shown in Figure 5D–F. These results reflect the *in vivo* tracking of SR-FLIVO-FNPs probe and the successful crossing of the weak compromised BBB due to vasogenic edema and the small size of the FNPs based probe used which can give a specific detection of apoptotic cells in the ischemic lesions.

**Figure 6:**
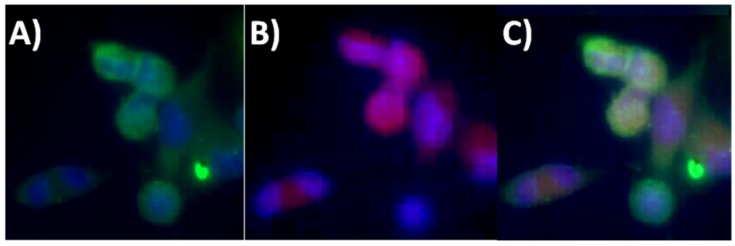
Fluorescence microscope images for apoptotic cells (**A**) TUNEL staining, (**B**) SR-FLIVO-FNPs staining and (**C**) merged TUNEL/SR-FLIVO-FNPs.

For *in vivo* intracellular tracking, Fluorescence microscope images shown in Figure 6 revealed that positive expression has been recorded using SR-FLIVO-FNPs probe at the ischemic core. This could be explained due to the covalent binding with caspase as previously reported [3,8].

**Figure 7:**
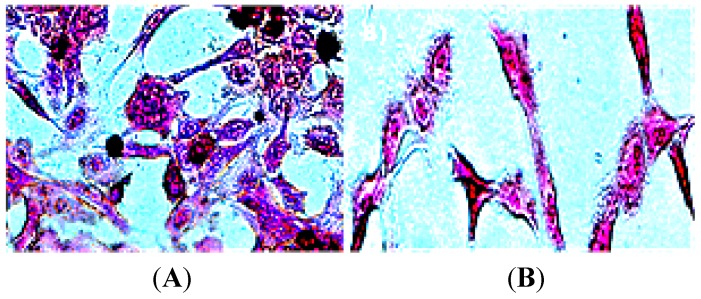
Light microscope images after Prussian blue staining in ischemic lesion (**A**) which shows the accumulation of SR-FLIVO-FNPs, and non-ischemic lesion (**B**) in which no remarkable accumulation of NPs was observed, respectively.

Moreover, Figure 7 shows the tracking and apoptotic cellular uptake of SR-FLIVO-FNPs probe after Prussian blue staining which confirms the intracellular probe uptake by apoptotic cells.

## 3. Experimental Section

### 3.1. Synthesis and Surface Modification of NPs

Fe_2_O_3_ nanoparticles have been synthesized using microemulsion procedure as previously described with some modifications [31]. In a typical synthesis method, 5.0 g of 0.15 mM FeCl_2_·4H_2_O was mixed with 0.5 mL of 1.0 M HCl at room temperature for 5 min (Solution A). 1.2 g CTAB was mixed with 10 mL *n*-Octane till a clear solution appeared followed by addition of 10 mL 1-butanol and stirring at 40 °C for 20 min (Solution B). Finally, solutions A and B were mixed together under vigorous stirring for 30 min at 40 °C followed by drop wisely addition of 6.0 mL of 0.25 M NaOH and kept under stirring for another 20 min to produce microemulsion of Fe_2_O_3_ nanoparticles. Decantation and then centrifugation carried out and finally the NP supernatant was washed thoroughly with water and acetone followed by drying.

For better stability, facile conjugation to other molecules, and inhibition of NPs aggregation, formation of silica shell surrounded the core of Fe_2_O_3_ NPs carried out via co-condensation synthesis method using a starting composition of equi-molar Na_2_SiO_3_·9H_2_O:FeCl_2_·4H_2_O as silica and iron sources, respectively. Removal of the hexadecyltrimethylammonium bromide (CTAB) surfactant carried out using hot ethanol solvent extraction method in which NPs (Fe_2_O_3_ and Fe_2_O_3_-SiO_2_) were immersed in hot ethanol at 50 °C for 20 h. Finally the surface grafting with NH_2_ group for Fe_2_O_3_-SiO_2_ NPs carried out using 3-Aminopropyltriethoxysilane (APTES) by immersing 1.0 g of the NPs in 20% APTES/Toluene mixture and allowed to interact for 12 h at 50 °C followed by washing thoroughly with ethanol and hexane.

Finally, the Fe_2_O_3_, Fe_2_O_3_-SiO_2_ and Fe_2_O_3_-SiO-NH_2_ (FNPs) were characterized using JEOL TEM (JEM 1400 Plus). For size measurements, a region of interest was chosen and sizes of 100 NPs or FNPs were statistically analyzed to calculate the final average size.

### 3.2. Conjugation of FNPs with SR-FLIVO

**Scheme 1:**
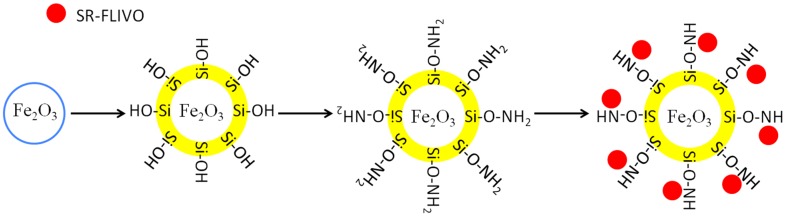
Schematic diagram for the synthesis and conjugation pathways of FNPs and SR-FLIVO.

Conjugation to SR-FLIVO has been carried out where 1.0 mg of FNPs was interacted with 1.0 mL of SR-FLIVO and the conjugation affinity has been recorded against time using HITACHI Fluorescence spectrometer (F-2500). Scheme 1 illustrates the synthesis and conjugation pathways of FNPs and SR-FLIVO.

### 3.3. In Vivo Study

A rat model of temporary focal cerebral ischemia has been used [32]. Spraque-Dawley rats (*n* = 4 for each group under study) underwent temporary middle cerebral occlusion for 2 h by using an intraluminal suture. Since the caspase activities and apoptotic cells were detected between 12 and 24 h after the reperfusion in pilot studies, SR-FLIVO-FNPs were injected 18 h after the reperfusion. The rats were allowed to survive for the next 60 min and then were sacrificed. The brains were fixed with formalin and sectioned into 50 μm thick serial slices. Histochemical TUNEL staining was performed and investigated with Olympus Fluorescence microscope (BX51).

In addition, TEM was recorded for the cellular tissue to ensure if; the SR-FLIVO-FNPs can passively cross the blood brain barrier (BBB) for apoptotic cells targeting. Moreover, Cellular Prussian blue staining was carried out to confirm the existence of SR-FLIVO-FNPs.

### 3.4. In Vitro Tracking

For *in vitro* tracking of SR-FLIVO-FNPs, the 9L gliosarcoma cell line has been used for cell culture study. For each well, the number of cells was adjusted to be 1 × 10^6^. Thus, 0.3 mM H_2_O_2_ has been used for *in vitro* apoptotic model preparation [33]. Therefore, cell morphology has been checked with time (Figure S1). An apoptotic model has been adjusted for 18 h then a solution of SR-FLIVO-FNPs was introduced to the apoptotic culture cells, and kept for additional 1 h, then washed thoroughly using D-PBS and finally TUNEL staining was performed followed by cellular recording using fluorescence microscope.

## 4. Conclusions

In summary, we have successfully developed a cell signaling probe that based on functionalized Fe_2_O_3_ NPs and fluorescent caspase inhibitor SR-FLIVO. This could be considered as a promising platform probe if it is well optimized to be used clinically as a contrasting agent in the future to specifically diagnose the apoptotic lesion and as a drug delivery vehicle; these potential uses are now under our consideration.

## Supplementary Materials

Supplementary materials can be accessed at: http://www.mdpi.com/2079-4991/5/2/874/s1.
