# Supplementary material for: Targeting of Apoptotic Cells Using Functionalized Fe2O3 Nanoparticles
Source: Nanomaterials (Basel). 2015 May 26;5(2):874–84. doi: 10.3390/nano5020874 (PMC5312913; doi:10.3390/nano5020874)
Supplement: Supplementary file 1 [file nanomaterials-05-00874-s001.pdf]

Supplementary Information

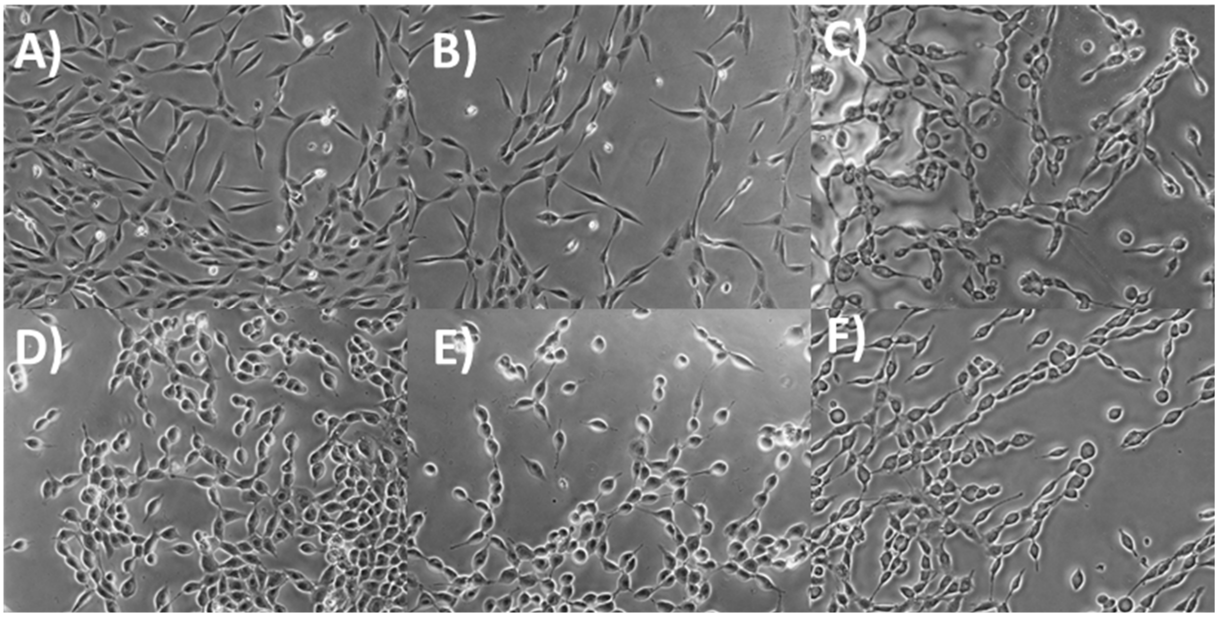

Figure S1. Morphological change with time for the 9 L glicosoma culture cell before addition of 0.3 mM H_2O_2 (**A**) and after addition of 0.3 mM H_2O_2, 6.0 h (**B**), 12.0 h (**C**), 18.0 h (**D**), 24.0 h, and 30.0 h, respectively.

© 2015 by the authors; licensee MDPI, Basel, Switzerland. This article is an open access article distributed under the terms and conditions of the Creative Commons Attribution license (http://creativecommons.org/licenses/by/4.0/).
